# Evaluating Current Density Modeling of Non-Invasive Eye and Brain Electrical Stimulation Using Phosphene Thresholds

**DOI:** 10.1109/TNSRE.2021.3120148

**Published:** 2021-10-28

**Authors:** B. A. Sabel, A. Kresinsky, L. Cárdenas-Morales, J. Haueisen, A. Hunold, M. Dannhauer, A. Antal

**Affiliations:** Institute of Medical Psychology, Otto-von-Guericke University of Magdeburg, 39106 Magdeburg, Germany; Center of Brain and Behavioral Sciences, 39106 Magdeburg, Germany; Institute of Medical Psychology, Otto-von-Guericke University of Magdeburg, 39106 Magdeburg, Germany; Institute of Medical Psychology, Otto-von-Guericke University of Magdeburg, 39106 Magdeburg, Germany; Institute of Biomedical Engineering and Informatics, TU Ilmenau, 98693 Ilmenau, Germany; Institute of Biomedical Engineering and Informatics, TU Ilmenau, 98693 Ilmenau, Germany; Center for Integrative Biomedical Computing, Scientific Computing and Imaging Institute, The University of Utah, Salt Lake City, UT 84112 USA; Department of Neurology, University Medical Center Göttingen, 37073 Göttingen, Germany

**Keywords:** Alternating current stimulation, current-flow modelling, phosphene, transorbital stimulation

## Abstract

Because current flow cannot be measured directly in the intact retina or brain, current density distribution models were developed to estimate it during magnetic or electrical stimulation. A paradigm is now needed to evaluate if current flow modeling can be related to physiologically meaningful signs of true current distribution in the human brain. We used phosphene threshold measurements (PTs) as surrogate markers of current-flow to determine if PTs, evoked by transcranial alternating current stimulation (tACS), can be matched with current density estimates generated by head model-based computer simulations. Healthy, male subjects (n=15) were subjected to three-staged PT measurements comparing six unilateral and one bilateral stimulation electrode montages according to the 10/20 system: Fp2-Suborbital right (So), Fp2-right shoulder (rS), Fp2-Cz, Fp2- O2, So-rS, Cz-F8 and F7-F8. The stimulation frequency was set at 16 Hz. Subjects were asked to report the appearance and localization of phosphenes in their visual field for every montage. Current density models were built using multi-modal imaging data of a standard brain, meshed with isotropic conductivities of different tissues of the head using the SimBio and SCIRun software packages. We observed that lower PTs were associated with higher simulated current levels in the unilateral montages of the model head, and shorter electrode distances to the eye had lower PTs. The lowest mean PT and the lowest variability were found in the F7-F8 montage (95±33 *μ*A). Our results confirm the hypothesis that phosphenes are primarily of retinal origin, and they provide the first in vivo evidence that computer models of current flow using head models are a valid tool to estimate real current flow in the human eye and brain.

## I. Introduction

Transcranial alternating current stimulation (tACS) is an emerging technique to alter brain functions by modulating brain oscillations [1]–[3]. One of its variants, transorbital ACS, is used in clinical applications for vision restoration in patients with optic nerve damage [4]–[8]. During delivery of tACS to healthy subjects or patients with visual system damage, the subjects experience the appearance of flickering lights, called phosphenes, a perceptual phenomenon that varies with stimulation frequency, intensity and electrode placement [9]. Phosphene thresholds (PTs) are often used as parameter to establish cortical excitability or to determine the stimulation intensity for a given treatment [7], [10]. Thus, the strength of phosphene perception is believed to functionally reflect the level of current strengths delivered by tACS, yet this assumption lacks verification.

Because true current flow cannot be directly measured in the intact eye or brain of healthy subjects, computational models were developed to simulate current flow and density distributions [11]–[14]. Such models may contribute to a better understanding of the origin and appearance of ACS induced phosphenes [15]. However, whether current density modelling accurately reflects the neurophysiological state of current distribution is still unclear. We, therefore, aimed to test this relationship by applying ACS with seven different electrode montages and correlating the PTs in a group of normal subjects with the computational modeling of current flow and current density distribution.

We considered the PT to be a functional marker of true current flow in the retina or central visual pathway, which can then be compared with estimated current flow as determined by computational modelling. The aim of our study was twofold: (i) to characterize how the delivery of tACS with different electrode montages influences PTs and (ii) to evaluate two current flow models by studying how current density estimations generated by head- and eye-model based computer simulations can be related to PTs, a functional marker of neuronal activation.

## II. Materials and Methods

### A. Subjects

Fifteen healthy male subjects were recruited (mean age: 23 ± 2.56 years, range: 21–31 years). Exclusion criteria were: non-corrected visual impairments, the presence of any disease of the eye or brain, electronic implants (such as heart pace-makers) in the body, metal implants in the head region (tooth implants were permissible) as well as recent consumption of alcohol, pharmaceutical or other illegal drugs or heavy smoking (>one pack/day). Every subject was informed about the risks, adverse effects and aims of this study before collecting their informed consent. The study was approved by the ethics committee of the Medical Faculty of Otto-von-Guericke University and was conducted according to the Declaration of Helsinki.

### B. Transcranial Alternating Current Stimulation (tACS)

tACS was delivered with a DC-Stimulator MC (NeuroConn, Ilmenau, Germany) and applied via two 3 × 3 cm^2^ rubber electrodes. The electrodes were positioned in saline-soaked sponges to ascertain good conductibility. Electrodes were fixed in seven different positions using an EEG-cap according to the international 10/20 system for electrode placements on the head. The position of the electrodes and the cable directions were carefully controlled and were exactly the same for a given montage. For suborbital fixation, we used Leukofix (BSN medical Hamburg, Germany) and a rubber band at the right shoulder. In order to reduce neurosensory side effects, impedance was kept below 10kΩ.

### C. Phosphene Threshold (PT) Measurement

PTs were measured in a three-step experiment, where the first two steps were needed to find the upper and lower borders of phosphene perceptions, and the third step determined the precise midpoint. The first measurement started after 10 min. of dark adaptation, the only light source was the monitor of the stimulator, which was positioned to face away from the subject. The subject sat on a chair facing a black wall, which covered the whole visual field. All stimulation bursts were performed with a frequency of 16 Hz based on the protocol used in a previous study [16]. Prior to the study, we determined that flickering phosphenes at 16 Hz are easier to locate than phosphenes flickering at other frequencies. Subjects were asked to keep their eyes open during the whole experiment, which was carried out in three steps:

#### First step:

ACS intensity was increased successively in 50*μ*A steps starting at 50 *μ*A up to 500 *μ*A until the subject reported phosphene perceptions (10 stimulation bursts). Each stimulation-burst lasted 10 sec (1 sec fade-in, 8 sec constant, 1 sec fade-out) with 10 sec inter-stimulation intervals.

#### Second step:

For each subject, the lowest intensity leading to phosphene perception in step one was taken as the reference value and current strength was then varied by −25 *μ*A, +25 *μ*A and +50 *μ*A relative to this reference. Each stimulation-burst was presented twice in step two (6 stimulation bursts). The inter-stimulation intervals were set at 20 sec. The lowest current value where the subject reported the perception of phosphenes twice was then used as the initial value in the third step.

#### Third step:

Stimulation-bursts were presented randomly with intensities ranging from −25 *μ*A to +25 *μ*A around the intensity determined in step two. These intensities were presented three times each. A control frequency at 5 Hz was added, which was also randomly presented once at these three intensities. These 12 stimulation-bursts were separated by a 30 sec inter-stimulus-interval. The stimulation intensity at which the subject reported appearance of phosphenes in at least two out of three trials was considered to be the final PT.

**Fig. 1.**
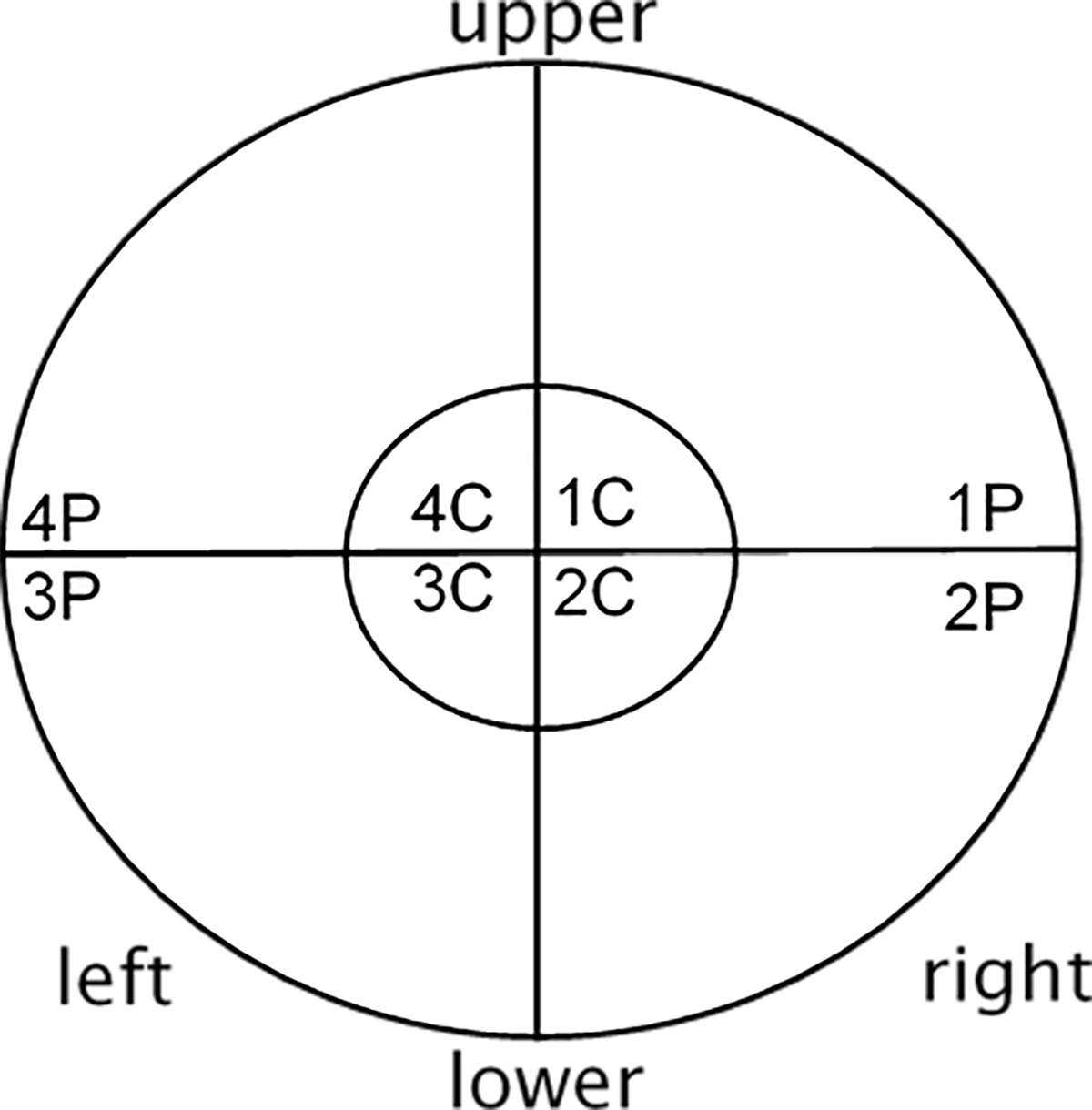
Visual Field Map; Subjects were instructed to relate the perception of the phosphene to this map; P = peripheral (0–5°), C = central (5–30°). Stimulation-bursts were presented randomly with intensities ranging from −25 *μ*A to +25 *μ*A around the PT of step two. These currents were presented three times each. After delivery of each stimulation-burst, participants were asked to report if they perceived phosphenes and where each phosphene was located within the visual field. The stimulation intensity at which the subject reported appearance of phosphenes in at least two out of three trials was considered to be the final PT.

After delivery of each stimulation-burst, participants were asked to report if they perceived phosphenes and to describe where each phosphene was located within the visual field (Fig. 1). The interval between PT measurements at different montages was 10 min. The total testing time was about 90 min. per day and testing was done on two different days with at least 24 hours between sessions to prevent adaptation of the brain to current stimulation.

**Fig. 2.**
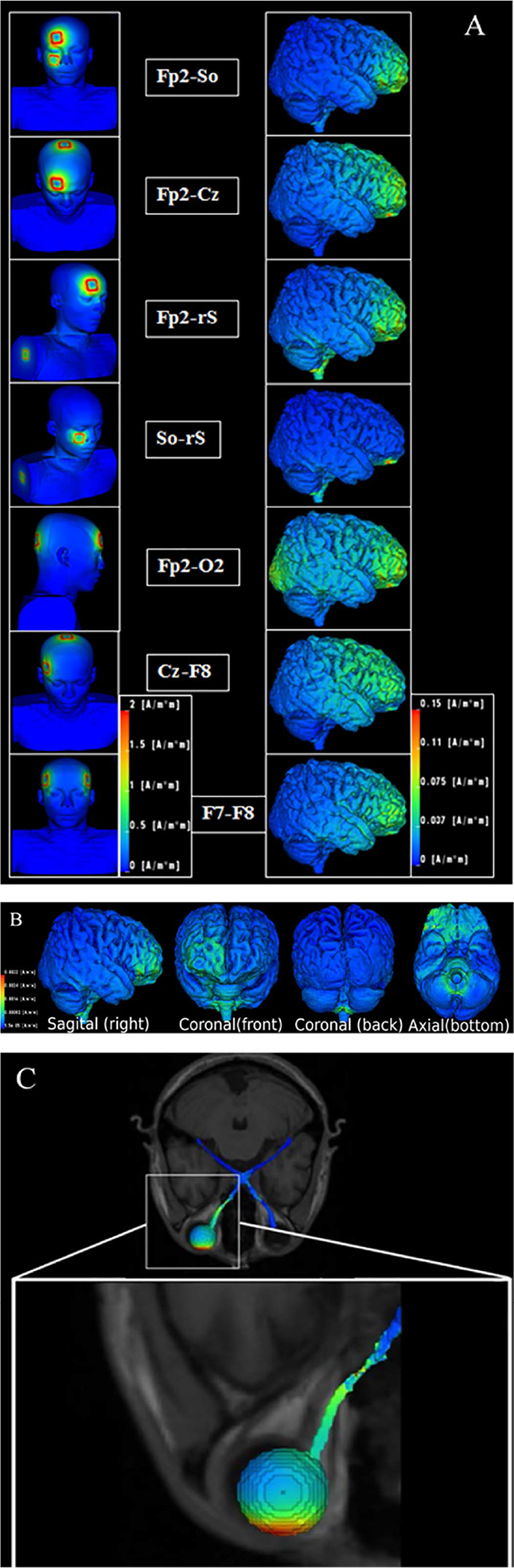
Part A: Visualization of seven electrode montages used (left) and calculated current density distribution in the brain. ACS was carried out with the following five eye-specific, unilateral montages: Fp2-Cz, Fp2-right shoulder (rS), Fp2-suborbital right (So), Fp2-O2, So-rS and the bilateral montage F7-F8. Additionally, we used the unilateral “not-eye-specific” montage Cz-F8. All electrodes were positioned according to the international 10/20 system EEG-electrode placement. Injected current: 0.5 mA. Part B: Brain current density distribution of the Fp2-rS montage from different perspectives show current flowing to the contralateral frontal cortex and passing along the base of the brain. Part C: in the Fp2-rS montage, current flows mainly through the right (ipsilateral) eye and optic nerve, but not to the left eye.

ACS was carried out with the unilateral montages Fp2-Cz, Fp2-right shoulder (rS), Fp2-suborbital right (So), Fp2-O2, So-rS and Cz-F8, and a bilateral montage F7- F8 (Fig. 2). All montages were positioned according to the international 10/20 system of EEG-electrode placement. The order of testing of the electrode montages was randomized between subjects.

### D. Alternating Current Flow Modelling

To visualize the distribution of the electric current flow of ACS, two different computer models, Model 1 and Model 2, were used for all seven montages (Fig. 2). Both were calculated with 3 × 3 cm^2^ electrodes and an injected current of 0.5 mA (model 1) and 1 mA (model 2), respectively. We labelled some montages to be “eye-specific”, i.e. those where the eye was located in or near the path of the main current flow: Fp2-Cz, Fp2-right shoulder (rS), Fp2-suborbital right (So), Fp2-O2, So-rS, and the bilateral F7-F8.

#### 1) Model 1 (“Brain Model”):

this was derived from two imaging data sets: (1) multi-modal imaging data (MRI and CT) of a head of a 40-years-old male [17], [18], and (2) Utah Torso Model [19] and carefully combined [20] to develop a finite element model of the head as well as the torso. Isotropic tissue conductivities like scalp (0.43 S/m), cerebrospinal fluid (1.79 S/m), brain (0.33 S/m), skull (0.01 S/m), electrode (1.5 S/m) and eye tissues (incl. optic nerve, 0.4 S/m) [17], [21], [22]. The torso was modelled homogenously as skin tissue conductivity to be able to place a shoulder electrode. The eyes as well as the optic nerve were modelled as one homogeneous tissue without modeling the retina explicitly due to constraints on computational resources. The complete electrode model (as described in Polydorides and Lionheart, Measurement Science and Technology, 2002) was applied on top of a 5 mm thick electrolyte surface (1.5 S/m) representing the electrode contact surface with an impedance of 10 kΩ.

Open-source software was used to generate a computational mesh (Cleaver 1.5.4) and to solve the quasi-static current injection problem (SCIRun 4.7, http://www.sci.utah.edu/cibc-software/scirun.html), which allows computing an estimate of the current density distribution of a one-time sample during tACS application when the cathode and anode reach its maximal current intensity value. Tetrahedral finite elements (Fes) with Robin boundary condition were used to model the injection of current at the electrode contact surface. The tetrahedral elements were generated using the Cleaver software package resulting in about 9/50 million nodes/tetrahedrons per montage model with min/average/max edge length of 0.2/0.6/1.4 mm (sampling depends on surface curvature) at the eye/optic nerve outer surface.

#### 2) Model 2 (“Retina Model”):

contained several tissue compartments modelling the eye (vitreous humor, lens, aqueous humor, retina, sclera, cornea, optic nerve), and surrounding muscles and fat. Each of these compartments was parameterized with a specific conductivity value. The model provides a more detailed view of the current density distribution in the retina and its vicinity. It was based on a structural T1-weighted magnetic resonance imaging (MRI) dataset using a 3D single-echo magnetization-prepared sequence from a healthy male volunteer (age 22) as acquired with a 3T MAGNETOM Trio (Siemens, Erlangen, Germany). MRI data were segmented using the FreeSurfer (FS) software (Martinos Center for Biomedical Imaging, Charlestown, MA, USA; http://surfer.nmr.mgh.harvard.edu) [23]. Based on the FS segmentation, binary masks for white and gray matter, cerebrospinal fluid, skull and scalp were extracted, using a segmentation approach [24] based on the Matlab (The Mathworks, Natick, USA) toolbox iso2mesh [25]. The different tissues surrounding the eye were segmented semi-automatically using in-house Matlab scripts applying iso2mesh functions. The combined binary masks were meshed with the freely available SimBio-Vgrid software (http://vgrid.simbio.de/) [26] to hexahedral FEs with inhomogeneous Neumann boundary condition at electrode nodes and homogenous boundary condition elsewhere. The stimulation current of 1 mA was equally distributed across the outer nodes of the electrodes.

We assigned the following conductivity values for the different tissue compartments: White matter (0.14 S/m), gray matter (0.33 S/m), cerebrospinal fluid (1.79 S/m), skull (0.014 S/m), scalp (0.33 S/m), vitreous humor (1.55 S/m), lens (0.32 S/m), aqueous humor (1.8 S/m), retina (0.7 S/m), sclera (0.56 S/m), cornea (0.5 S/m), optic nerve (0.03 S/m), muscle (0.35 S/m), fat (0.04 S/m) [27]–[32]. The electrode model was a 4 mm thick electrolyte layer with 1.4 S/m conductivity [33].

We obtained the scalar electric potential at each node of the mesh and calculated the vectorial current density in each finite element generated by tACS. Finite elements were directly derived from MRI voxels with 1 mm isotropic spatial resolution.

### E. Statistical Analysis

For statistical analysis, we used IBM SPSS STATISTICS 24. Nonparametric analyses used Friedman’s two-factorial analysis of variance for dependent samples and a post-hoc pairwise comparison using the Wilcoxon signed-rank test with a Bonferroni correction for multiple comparisons. The significance level was set at p = 0.05. The Mann-Whitney U test for independent samples was used to analyze the impact of the eye dominance on the PT. PTs and simulated current densities were then correlated using a one-tailed Spearman rank correlation. Concerning Model 1, we used the mean current density magnitude on the outer surface for left / right eyeball as well as different brain ROIs. With regard to Model 2, the mean current density magnitude of elements representing the retina (posterior spherical semi-shell of the eyeball) was correlated with threshold current.

To determine the impact of the electrode montage on phosphene perception, we calculated Pearson correlation coefficients between electrode distances to the eye or the occipital cortex and PTs. To this end we determined for each montage the average distance (in cm) of both electrodes to the eye or the occipital cortex, taking these averages as a surrogate for the influence of the respective montage has on the PTs. These distance values of the montages were then correlated with the respective PTs (the So-rS montage was excluded from this analysis because of its low current values in the eye and the occipital cortex).

## III. Results

In the first step, we measured PTs using different electrode montages to identify the montage that could most easily elicit phosphenes. In the second step, we compared the two different models of current distribution to study if and how current densities in different regions of the eye and the visual pathway match with subjective phosphene thresholds. We reasoned that if simulation methods of current flow reflect true current flow, then an inverse relationship should exist in that higher current densities in the eye correlate with lower PTs. In other words, we predicted that phosphenes could be elicited more easily, even at lower current strength, with “eye-specific” montages where the eye is positioned inside or near the main current path. Of note, even montages with occipital electrodes are expected to produce significant current flow to the retina during stimulation (see [15]).

**Fig. 3.**
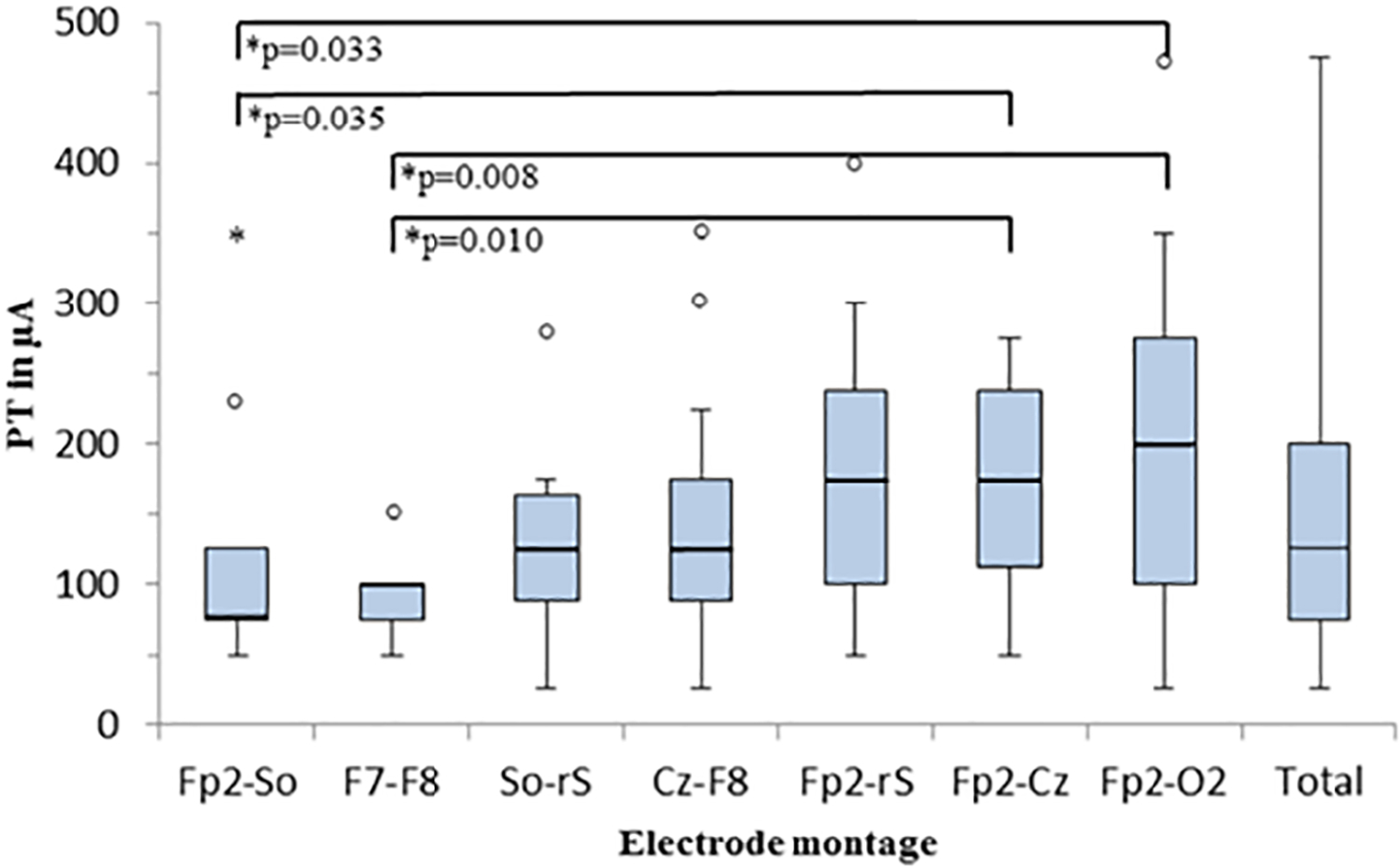
The median and range of PTs in *μ*A at different electrode montages. The Bonferroni-corrected Wilcoxon signed-rank test showed significant differences between F7-F8/Fp2-O2 (p = 0.008), F7-F8/Fp2-Cz (p = 0.010) and Fp2-So/Fp2-O2 (p = 0.033), and Fp2-So/Fp2-Cz (p = 0.035) electrode montages.

PTs for different montages are displayed in Tab. I. The lowest PT and the lowest variability was found in the F7-F8 montage with a mean current density strength at phosphene threshold of 95±33 *μ*A (mean±SD) and the highest PT with the Fp2-O2 montage (187±107 *μ*A). Fig. 3 shows the boxplot of median and range of PTs and significant differences. The Bonferroni-corrected Wilcoxon signed-rank test showed significant differences between F7-F8/Fp2-O2 (p = 0.008), F7- F8/Fp2-Cz (p = 0.010) and Fp2-So/Fp2-O2 (p = 0.033), and Fp2- So/Fp2-Cz (p = 0.035).

### A. Probing Phosphene Adaptation and Adverse Events

Because the PT experiments were conducted on two independent days of testing, which were at least 48 hours apart, we checked if PTs are different on the second day because of possible perceptual learning effects caused by repetitive tACS. The comparison of the median differences between both days using the Wilcoxon signed-rank test showed no significant change over time, though there was a statistical trend of an increasing PTs on day 2 (z = −1.669; p = 0.096). This is a sign that some adaptation may have taken place, which is, in fact, routinely observed when using ACS in clinical contexts (unpublished observation). Nevertheless, other (between-subject) factors might contribute to variability of phosphene perception and reporting, such as changes or criteria for determining threshold, stimulus duration, ambient light or dark adaptation [15]. Yet, these factors are expected to be minimal during within-subject testing.

All subjects tolerated the stimulation well and no adverse events were reported.

### B. Eye Dominance

The Mann-Whitney U Test for independent samples showed no significant influence of eye dominance on PT (p = 0.38).

### C. Correlation of Current Density Modelling and PTs

**Fig. 4.**
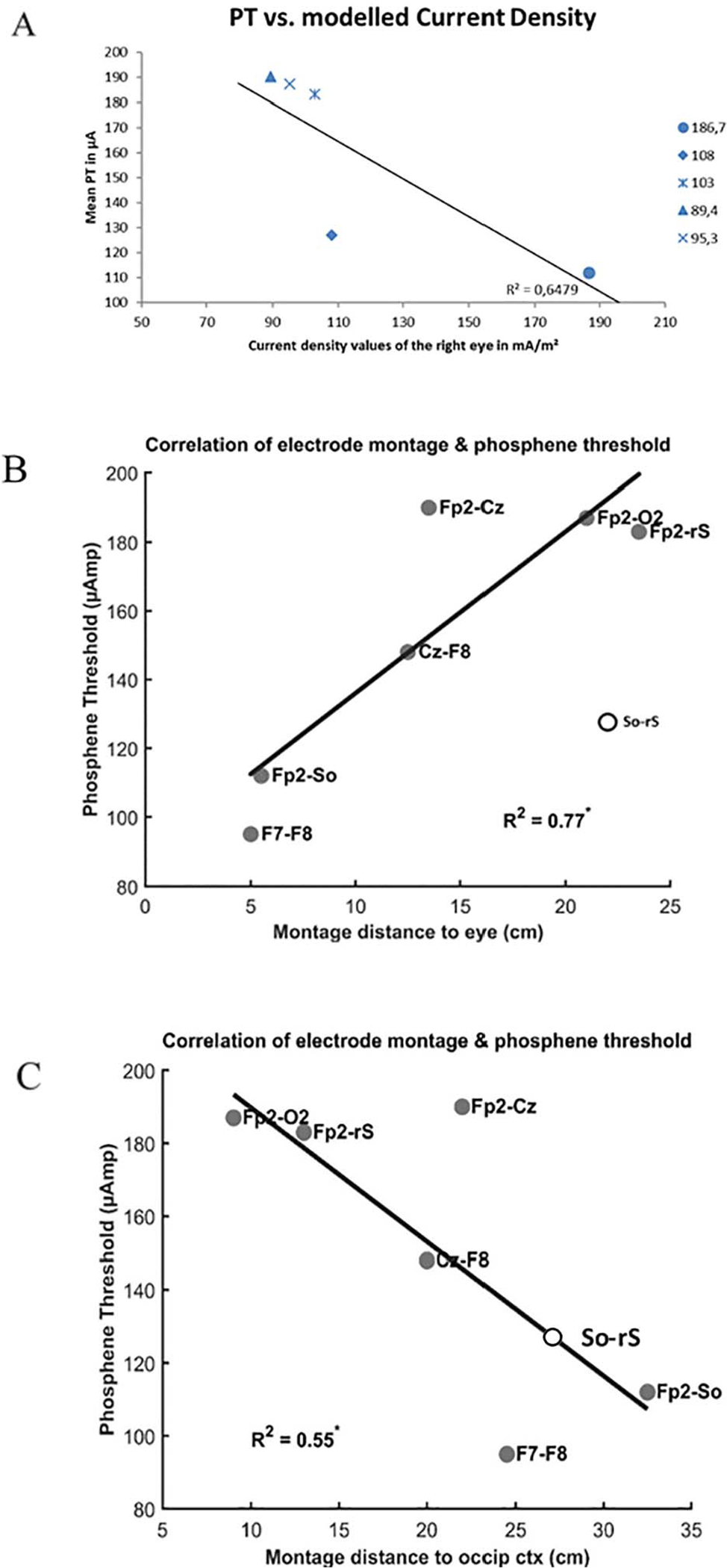
*Panel A* shows the mean PTs and modelled current density values of the eye-specific montages. A negative relationship was observed between the mean PTs of the eye-specific, unilateral electrode montages (Fp2-So: 186.7; So-rS: 108; Fp2-rS: 103; Fp2-Cz: 89.4; Fp2-O2: 95.4) and the calculated current densities for these montages (see also Tab. I and II). *Panel B and C* display the phosphene threshold as a function of electrode distance from the eye and occipital cortex for each montage, respectively. The average distance of both electrodes in cm represents a surrogate for the influence a montage has on the PTs. Lower phosphene thresholds were significantly correlated with shorter distances to the eye (r2 = 0.77, p < 0.01) and longer distances to the occipital cortex (r2 = 0.55, p < 0.05) (So-rS montage was not included in the correlation analysis; see methods).

Tab. I shows the estimated current density values as obtained by modelling for one (right) eye for each montage and the median PTs. Both simulation models were rather comparable. However, a difference between the models was found in that suborbital-right shoulder montage with 30.3 mA/m^2^ and the Fp2-Suborbital montage with a difference of 48.6 mA/m^2^. Highest current density values were found for the Fp2-So electrode montage in both models. While a one-tailed correlation between mean PTs of all seven electrode montages and the simulated current densities of the right eye in the eye-specific modelling was not significant, a negative relationship was observed between current density and mean PTs of the eye-specific, unilateral electrode montages (see regression line in Fig 4a, r2 = 0.65, n.s.). Imporantly, lower phosphene thresholds were significantly correlated with shorter electrode distances to the eye (r2 = 0.77, p < 0.01) and longer distances to the occipital cortex (r2 = 0.55, p < 0.05; Fig 4b,c).

### D. Comparison of Phosphene Localization and Simulated Retinal Current Distribution

**Fig. 5.**
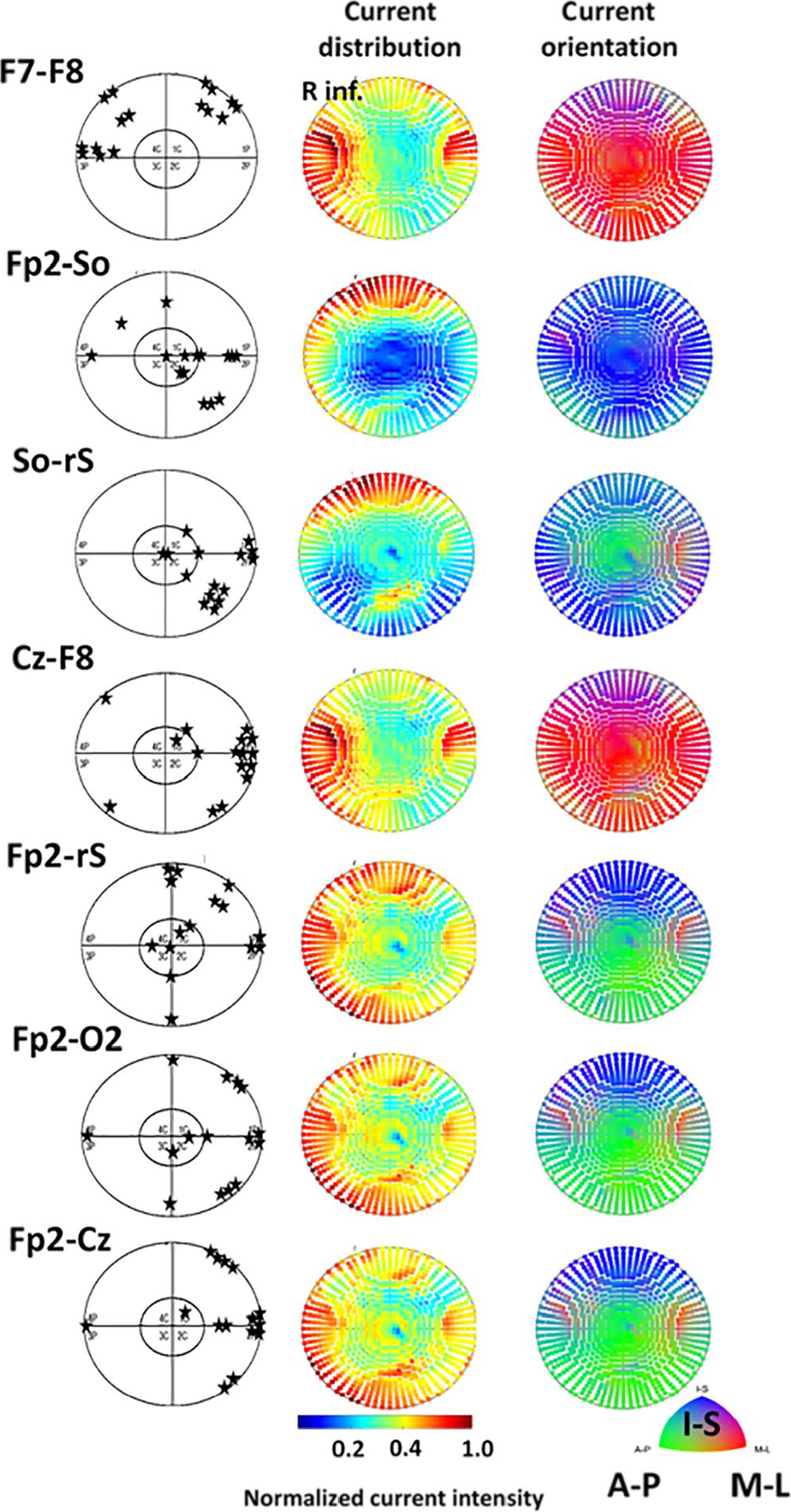
Localization of reported phosphenes (indicated by a star) within the visual field (left) and current density distribution (middle) and the current orientation (right) in the retina for the seven electrode montages. Current simulation was maps were mirrored to match visual fields and simulation maps (e.g. left superior visual field and right inferior retina). Every star in the visual field maps represents localization of phosphene of one subject. The topographic distribution shows no obvious association of current distribution or orientation of different montages and phosphene perceptions. Yet, the large majority of phosphenes were reported in the periphery, which roughly corresponded with the current density simulations: higher current densities were predicted peripherally and lower densities in central areas.

Fig. 5 shows (i) the localization of the phosphenes as reported by the subjects and (ii) current densities and current orientations the of the retina simulation. For ease of interpretation, the retinal maps were flipped in both dimensions to match the visual fields, e.g. the retinal lower left quadrant is displayed on the upper right quadrant to match the visual field. The topographic distribution shows that higher values of retinal current densities in the left and right periphery of the ventral retina roughly matched the peripheral phosphene locations in the upper half of the visual field; in fact, this was reported by every subject (Fig. 5**a**). Not surprisingly, in the bilateral electrode montage the current flow model could precisely predict the localization of the phosphenes. However, for the monocular montages (for example see Fig. 5 **b**, **c**) we did not find such tight correspondence between current flow modelling and phosphene perception. Yet, the large majority of phosphenes were reported in the periphery, which roughly corresponds with the current density simulations. Here, we always observed higher current densities peripherally and lower current densities in central areas.

### E. Current Density Simulation of Different Brain Areas

Tab. II shows for both brain hemispheres the current density values for the lateral geniculate nucleus (LGN) and the occipital cortex (OC) for a modelled current injection of 0.5 mA. While we found no significant correlation between PTs and simulated current densities of the LGN, the occipital cortex current density correlated significantly and positively with PTs for all seven montages (Spearman rank correlation coefficients: r_s = 0.929, p = 0.001). As expected, the occipital cortex current density in the Fp2-O2 montage was higher than the montages with no occipital electrode. But in contrast to our other observation, we observed here that very high current density values were associated with the highest PTs. Fig. 2 shows cortical current density values as visualized by the simulations. Most montages showed very low occipital current densities, while pronounced current densities were found in frontal areas. This supports the hypothesis that phosphenes evoked by transorbital ACS do not have an occipital origin or that the PT is much higher so that retinal ones will always be elicited earlier via current conduction effects.

## IV. Discussion

The present study was designed to investigate if current flow models are useful estimates of real current flow. To this end, we used the visual system as a model to relate alternating current density to PTs as a possible functional surrogate of neuronal activation. The underlying assumptions were that greater neuronal activation is a direct function of greater current density during ACS and that this can be probed by lower PTs. We reasoned that if there is a clear association between current densities as estimated by computer-simulation modelling and PTs, this could inform us if current flow modelling is a good approximation of true current flow.

We have used two different simulation models. The chosen conductivity values were selected based on prior reports in the literature. The uncertainty in conductivity values is reflected in the differences in predicted electric currents and partially can explain these. Generally, there are no published simulation studies that use the same models. Therefore, a comparison of two different models was deemed to strengthen our final conclusions. Specifically, we observed (i) that lowest ACS evoked PTs were recorded using frontal montages, especially in the F7-F8 and Fp2-So montages, with significant differences to the occipital montage Fp2-O2, (ii) a significant negative correlation between the modelled current densities and the measured PT intensity in the eye specific electrode montages, and (iii) there was an association between high peripheral retinal current density and the perception of peripheral phosphenes, i.e. the current flow roughly matched phosphene localization.

In line with previous studies [10], [16], [34], [35] we found a strong relationship between electrode positioning and phosphene perception, lower PTs in frontal and higher PTs in occipital montages. In other words, electrodes closer to the eye elicit phosphenes during AC-stimulation more easily than those positioned further away. In contrast to prior studies, we used monocular montages (and one bilateral montage), which are more specifically aimed at stimulating the eyes. Our aim was to check if even very low current intensities can be used to elicit phosphenes. Indeed, of all montages, the “eye-specific” montages Fp2-So, So-rS and F7-F8 showed the lowest PTs, and the F7-F8 montage was the most effective one with also the smallest variability. They are therefore the most feasible montage for clinical applications in patients with disorders of the retina and optic nerve [4]–[8].

The observation that the lowest PTs were found in frontal montages and the highest PTs in Fp2-O2 is compatible with the hypothesis that the origin of ACS-induced phosphenes is primarily retinal, not cortical [15]. In fact, we found that a closer distance of the electrodes to the eye significantly correlates with a lower phosphene threshold (Fig 4b). In contrast, PTs are highest when the montage is positioned at or near the occipital cortex (Fig 4c). How can this be explained? We suggest that current can reach the eyes from any electrode position on the skull. It can easily shunt through the skin tissue of the head with its relatively low resistance compared to the high resistance of the bony skull. Our observations are therefore compatible with the concept that at a given current strength, more current flows towards the eye (through the skin) than (through the skull) to the occipital cortex. Hence, current shunting to the eyes is highly likely in most (if not all) montages. This interpretation of our results is in agreement with the computational results by Laakso and Hirata [15] who - on theoretical grounds - proposed that even electrodes distant from the eyes can produce considerable current flow to the retina. Our results are an empirical confirmation of this prediction. In agreement with these authors we too believe that threshold current densities are not only influenced by the electrode montages but also by other experimental conditions such as different criteria for determining threshold, duration of stimulation level of observer training, introspection ability, adaptation phenomena, normal vs. abnormal visual system function and so on. While all of these factors contribute to the variance of results, the location and size of the electrodes are probably among the dominating factors.

This interpretation is also supported by the significant positive correlation of PTs and simulated current densities at the occipital cortex. The correlation shows that occipital electrode positioning, which is the only way to reach higher occipital current densities, results in higher PTs. Accordingly, negative correlation between PTs and modelled current densities of the right eye and retina was also observed, indicating that phosphenes can be obtained with less current when stimulating regions near the eye. We found no significant impact of the eye dominance on the PT, suggesting again that the underlying mechanism of the phosphenes is probably the activation of retinal ganglion cells as proposed earlier [36]–[38]. Besides the well-known frequency-dependent effects of tACS on phosphene perception, a recent study demonstrated phase-dependent interference effects of them, consistent with the changes of the current density in the retina [37]. Though it is known that phosphenes can be elicited by the visual cortex stimulation using TMS [39], this is not yet proven, only suggested for tACS [9]. In contrast, stimulation of the eyes can be done using much lower current intensities because they are not protected by bony structures, allowing much lower current strength to elicit phosphenes than montages aimed at the brain, where currents have to traverse the skin, subdermal tissue and skull. Thus, it is expected to be difficult (though theoretically possible [15]) to elicit cortical phosphenes using tACS with occipital montages without unintentionally stimulating also the eye. These considerations are in accordance with previous findings [15], [40]. The question is still open if phosphenes can be produced by stimulation of the occipital cortex alone using tACS, an issue to be studied further.

### A. Evaluating Current Modelling

The most fundamental conclusion of the present study is that, for the first time, we obtained evidence that current density modelling provides a realistic estimation for tACS-induced current flow and related phosphene perception (at least for the two models we studied). In earlier current flow modelling studies multi-electrode arrays or disc electrodes were used for electrical stimulation of the retina with the aim to develop prosthetic devices [41], [42]. In our study, we found high correlations between modelled current densities and functional PT parameters: lower PTs strongly correlated with higher current densities of the right eye across all measured montages and significant correlations for eye-specific montages. Additionally, a significant positive correlation was observed between higher PTs and greater current densities of the occipital cortex, confirming the findings by Laakso and Hirata [15].

Regarding the phosphene localization and the peak values of current distribution in the right retina, a correlation was found between high peripheral current density values and also most phosphene perceptions in the periphery of the visual field. However, forecasting phosphene perception by using retinal maps with current density distribution is still imprecise. Highest current densities for reported phosphene localization were expectedly found at the opposite side of the theoretical retinal activation. This was observed for all unilateral electrode montages. In contrast, the high F7-F8 current densities of the inferior retina and the superior localization of phosphenes matched rather well. Nevertheless, because of the high inter-subject variability of phosphene perception (which are subjective reports), it is difficult to establish a clear one-to-one relationship.

How to reduce this variability, e.g. by using montages close to the eyes like Fp2-So or by improving the subjects’ ability to report phosphenes, is an issue deserving further studies. Of note, we are not proposing that the origin of phosphenes is only retinal but phosphenes can also be elicited by cortical activation [43], and there are montages studied by Laakso and Hirata [15] that help prevent unintended retinal stimulation. Yet, in agreement with their computation modelling results we showed in normal subjects that phosphene perception correlates indeed with current densities modelling in the retina and brain. While Laakso and Hirata used computational modelling to study which montages produce (unwanted) retinal stimulation effects, our goal was to find montages that maximize current density in the eye to so optimize existing protocols for clinical applications of vision restoration [4]–[8].

While it is not possible to precisely predict the location of phosphenes based on retinal current density modelling, we found that higher peripheral retinal current densities correlate with the subjective reporting of peripheral phosphenes.

We conclude that with our kind of analysis it is not possible to precisely predict the localization of perceived phosphenes from computational simulations of the retina for unilateral montages; yet, the greater response of the peripheral retinal modelling and peripheral phosphene perception’ threshold matched as well. To help improve the association of PT and current flow simulation, future studies could consider the known relationship between the orientation of current flow and sensitivity to stimulation: it is conceivable that low central current density producing phosphenes may be driven by increased sensitivity at that location, if current flow is indeed orthogonal to the retina [15].

In summary, because the topography of the retinal current flow generally matches the topography of the phosphene perception, this is compatible with the hypothesis that – on the retinal level –current flow modelling is a very good reflection (or partial validation) of “real” current flow, probed here with PT as a surrogate of neuronal activation. However, the nature of the phosphenes regarding size, color, shape etc. is subjective and very different between subjects and does not lend itself easily to systematic analysis.

## V. Limitations

PT was measured only in young subjects. Further studies are required to better characterize phosphene perception across the lifespan of healthy volunteers and compare them to a clinical population. A second limitation is that we measured PTs with both eyes and specific details with each eye were not explored; yet it seems that separate PT measurements for right and left eye are not needed since eye dominance did not influence our results. We could evaluate two different brain current flow models using different software and tissue models parameterized by slightly different sets of literature-based conductivity values (e.g., scalp). Hence, estimations of current flow provide only relative and not absolute current values. It is also conceivable that orientation of current densities, i.e. splitting the currents into normal vs. tangential components, might provide more precise correlations and phosphene distributions, which could be explored in future studies, and future studies would benefit from more detailed analysis of coronal and axial views of current flow in the brain.

Another limitation is that Model 2 aims at specific compartments of the retina. This model was derived from MRI data with 1 mm isotropic resolution. When transferring this resolution to the hexahedral finite element model, the retina was approximated as the outer elements of the posterior spherical semi-shell of the eyeball, leading to an over-estimation of retinal thickness. Yet another limitation of our study is that the scalp conductivities are different in models 1 and 2 and results can therefore not be directly compared. It is therefore recommended to determine accurate conductivities when different models are compared. In future studies, more detailed models (e.g. of the retina) can be realized when MRI data with higher resolution are available to study ACS effects [44], [45], and we should explore in greater detail the role of the direction of current flow as a factor of phosphene threshold/sensitivity. Finally, whether there is an added value to image each subject’s brain individually to adjust electrode montages is still unclear and needs further exploration.

## VI. Conclusion

Our study shows that computer-based simulations of current flow in the human eye and brain can be matched with functional parameters, such as PT measurements. In this way, current density simulation helps us to better understand the mechanisms of phosphene perception induced by ACS, as it provides in vivo confirmation of earlier computational evidence [15] that phosphenes are predominantly of retinal, not cortical, origin. In sum, our observations are compatible with the proposal that computer simulations of current flow using head models are useful tools to estimate true current flow in the human eye and brain.

## Figures and Tables

**TABLE I:** PTs and Simulated Average Current Densities of the Right Eye for Both Modelling Methods. PT Values Are Given in *μ*A. Current Density Estimates as Calculated by Modelling Are Given in mA/m^2^

Electrode montage	Phosphene Threshold Intensities		Eye Current Density	
	Median	Mean ± SD	Brain model	Eye model
Fp2-So	75	112 ± 80	138.1	186.7
F7-F8	100	95 ± 33	43.1	38.5
So-rS	125	127 ± 64	77.7	108.0
Cz-F8	125	148 ± 89	41.4	32.5
Fp2-rS	175	183 ± 104	100.7	103.0
Fp2-Cz	175	190 ± 108	98.6	89.4
Fp2-O2	200	187 ± 107	100.2	95.3

**TABLE II:** Simulated Current Density Distribution in the Visual Pathway. LGN = Lateral Geniculate Nucleus, OC = Occipital Cortex. Current Densities Are Given in mA/m^2^. PT Values Are Given in *μ*A. Injected Current: 0.5 mA

Electrode Montage	PT medians	right LGN	left LGN	right OC	left OC
Fp2-So	75	16.9	18.8	10.2	10.1
F7-F8	100	24.0	18.7	10.9	8.2
So-rS	125	8.7	17.4	11.0	11.3
Cz-F8	125	35.5	16.0	19.7	19.6
Fp2-Cz	175	24.1	15.8	17.5	17.5
Fp2-rS	175	24.8	36.5	21.2	21.5
Fp2-O2	200	19.8	21.8	79.8	42.0
